# Manufacturing and Characterization of Composite Fibreboards with *Posidonia oceanica* Wastes with an Environmentally-Friendly Binder from Epoxy Resin

**DOI:** 10.3390/ma11010035

**Published:** 2017-12-26

**Authors:** Daniel Garcia-Garcia, Luis Quiles-Carrillo, Nestor Montanes, Vicent Fombuena, Rafael Balart

**Affiliations:** Instituto de Tecnología de Materiales-ITM, Universitat Politècnica de València-UPV, Plaza Ferrándiz y Carbonell 1, 03801 Alcoy, Spain; dagarga4@epsa.upv.es (D.G.-G.); luiquic1@epsa.upv.es (L.Q.-C.); nesmonmu@upvnet.upv.es (N.M.); vifombor@upvnet.upv.es (V.F.)

**Keywords:** biobased epoxy, fiber boards, waste management, *Posidonia oceanica*, hot-press manufacturing, coupling agents

## Abstract

Highly environmentally-friendly fibreboards were manufactured by hot-press moulding using *Posidonia ocaeanica* wastes and a partially biobased epoxy resin as binder. Fibreboards with a constant fibre content of 70 wt % were successfully manufactured by thermo-compression. The effects of a conventional alkali treatment were compared to the synergistic effects that additional silanization with two silanes (amino and glycidyl) can exert on the mechanical and thermo-mechanical properties of fibreboards. The results revealed a remarkable improvement of the mechanical properties with the combination of the alkali treatment followed by the silanization. Scanning electron microscopy also revealed increased resin-fibre interactions due to the synergistic effect of both amino- and glycidyl-silanes. These fibreboards represent a formaldehyde-free solution and can positively contribute to sustainable development as the lignocellulosic component is a waste and the binder resin is partially biobased.

## 1. Introduction

Nowadays most developed countries are paying special attention to environmental issues. Some of the most important actions to protect the environment are focused on the optimum use of natural resources, the reduction of polluting gas emissions, upgrading industrial and/or agroforestry wastes, etc.; all this with the main aim of reducing the carbon footprint and positively contributing to sustainable development based on a circular economy concept. Fossil fuels are widely used as the main source for many applications, including the polymer and composites industries. Petroleum depletion is also acting as the leading force to the development of a new series of environmentally-friendly materials from renewable resources. This environmental sensitivity is particularly marked in the polymer and polymer-composite industries, which, traditionally, are highly dependent on petroleum-derived polymers and resins. With regard to thermosetting resins, petroleum-derived epoxies, unsaturated polyesters, vinyl esters, phenolics, acrylics, etc., still represent the main source of industrial resins for uses in the composites industry and for the manufacturing of fibre and particle boards.

Particle and fibreboards are widely used in the building industry as eco-friendly solutions to wood with increasing uses in thermal insulators, ceiling boards, wall partitions, etc. due to an excellent combination of mechanical, thermal and acoustic properties together with a competitive price. Particle and fibreboards consist of a major lignocellulosic component bonded with a resin, typically, phenol-formaldehyde (PF), urea-formaldehyde (UF), melamine-urea-formaldehyde (MUF) [1], by hot press moulding [2]. Despite wood being one of the most relevant lignocellulosic components in particle/fibreboards [3], new lignocellulosic particles and fibres (mainly by-products from other industries) have been successfully introduced into these boards, i.e., corncob, sawdust [2] date palm branches [4], sorghum stalk fibres [5], almond shell and rice husk [6], etc. As reported by Vaisanen et al. natural fibre polymer composites (NFPCs) represent a technical and economical solution to upgrading agricultural and forest wastes [7].

With regard to the binder, some recycled thermoplastics have been proposed as binders in wood plastic composites and particle boards [8]. The current tendency is to avoid formaldehyde as it is considered a great pollutant [1], so that new formaldehyde-free binders are continuously being investigated. Most of them are derived from tannins [9,10], lignin [11,12], soy protein [13,14,15], etc. Although most of the commercial epoxies come from petroleum, in the last years, new environmentally-friendly epoxies from vegetable oils have been proposed as eco-friendly adhesives and composite matrices [16,17,18]. Obradovic et al. reported the potential of acrylated epoxidized soybean oil (AESO) in producing industrial foams with cellulose reinforcements [19]. Even more, Arevalo et al. have proposed the use of the self-binding capability of cellulose to obtain binderless fibreboards by hot-pressing [20]. Kojima et al. also explored the potential binding effects of lignocellulose nanofibers (LCNF) with wood flour to give composite boards with interesting properties [21].

*Posidonia oceanica* (PO) is an endemic seagrass of the Mediterranean Sea. It plays a key role in coast preservation, as it is present on seabeds and positively contributes to the delay or minimization of erosion effects. After strong storms, the whole plants or parts (stems, leaves) are torn off the seabed and move towards the shore. Consequently, huge amounts of this waste accumulates along many coastal beaches thus leading to an important visual impact together with intense odours due to decomposition. The coastal municipalities must periodically remove this waste to obtain the appropriate quality seals, such as the blue flag and others, which play a key role in attracting tourism. Over the last years, new uses for this waste has been proposed. Some authors have reported the potential of PO in the filtration and removal of different pollutants [22,23,24,25,26,27]. Due to its high cellulose content, some authors have focused their research on obtaining cellulose derivatives such as nanofibrillar cellulose, nanocrystals, cellulose acetate, glycidyl methacrylate grafted cellulose, etc. and their potential as reinforcements in different matrices [28,29,30,31,32,33,34]. It is worth mentioning the work of Cocozza et al. which reports the alternative use of PO wastes as a compost material or for energy recovery [35]. PO wastes have been used as a reinforcement into several polymer matrices such as polyethylene [36,37], polyhydroxyalkanoates (PHAs) [38], processed by extrusion with PO contents up to 40 wt %. With regard to the use of PO in particleboards, Maciá et al. have recently reported the partial substitution of pinewood by PO in particleboards with polyurethane binders with interesting applications in the building industry [39]. Our previous work with PO showed the technical viability of fibreboards containing gluten as a biobased binder in the 10–40 wt % range, processed by hot press moulding, but the impact toughness was poor [40]. 

This work is focused on the development of fibreboards with PO fibres using a partially biobased epoxy as the binder resin. With the aim of enhancing the impact toughness, different surface treatments on PO fibres are assessed.

## 2. Materials and Methods

### 2.1. Materials

**Figure 1:**
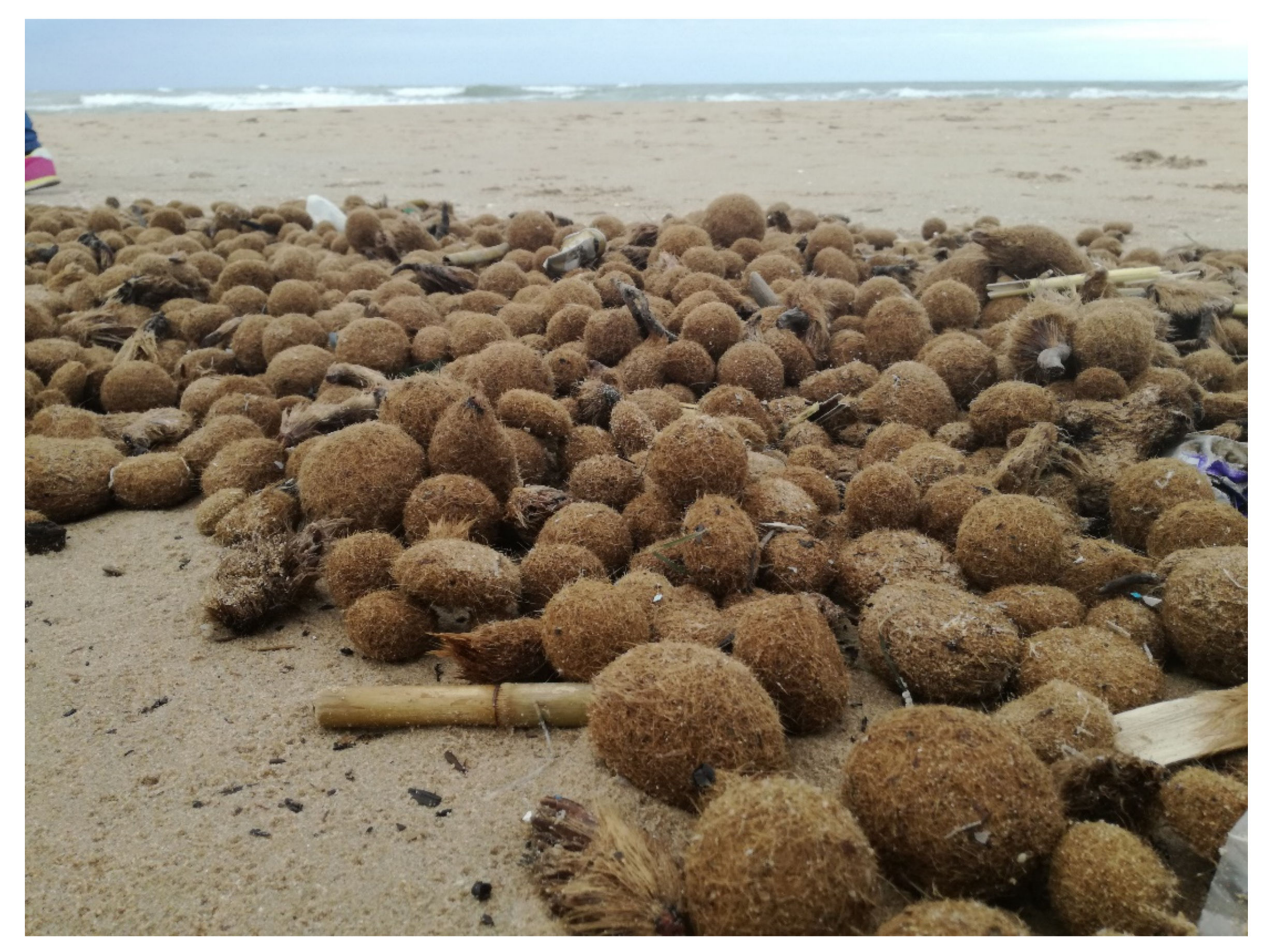
Image of “*Posidonia oceanica*” balls collected from the Mediterranean seashore.

**Scheme 1:**
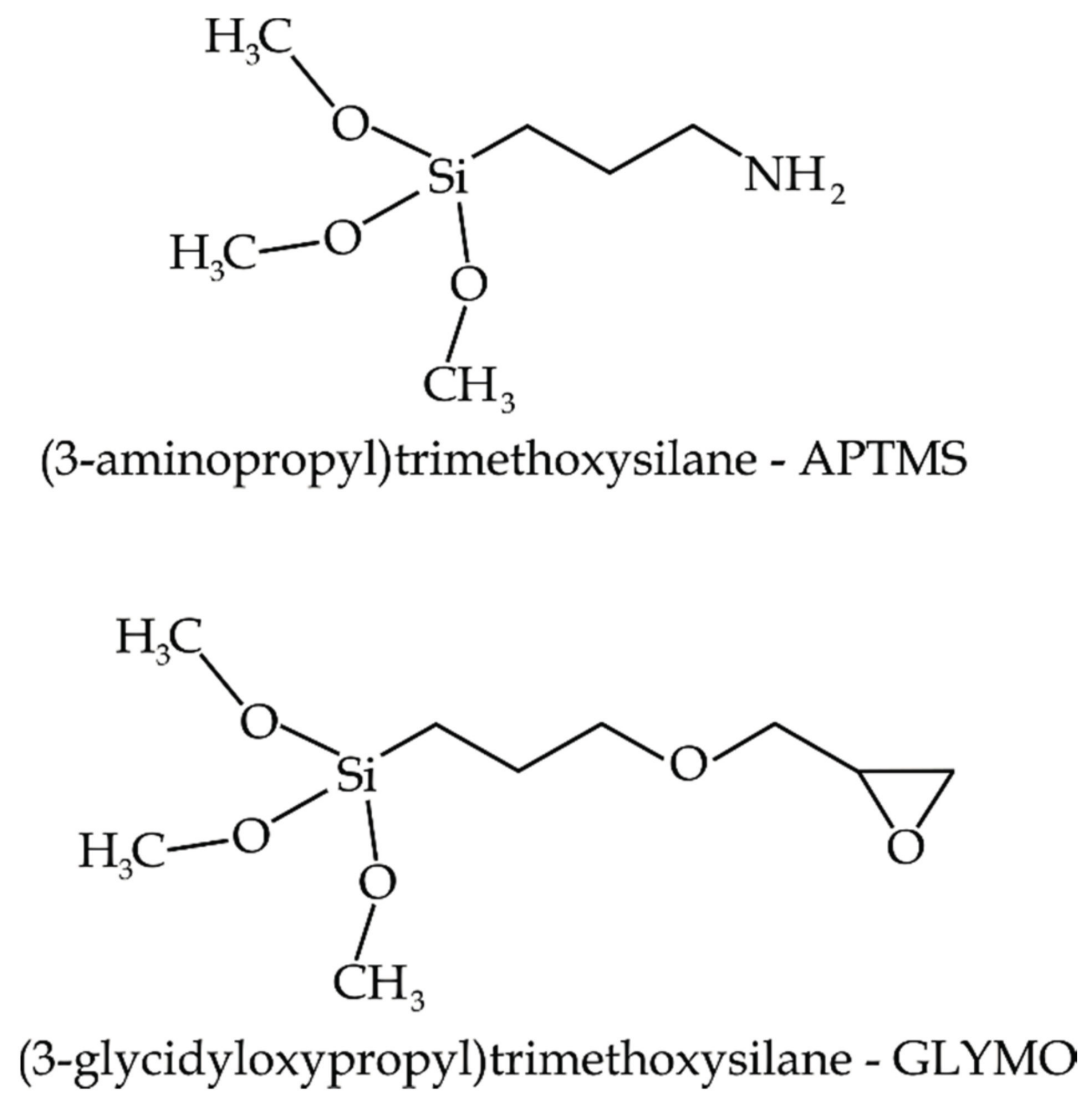
Schematic representation of the chemical structure of the silane-coupling agents.

*Posidonia oceanica* (PO) balls (Figure 1) were collected from the Mediterranean coast. These balls were ground to a fibre size comprised between 2 and 8 mm length. Sodium hydroxide (99%) was supplied in flake form by Cofarcas S.A. (Burgos, Spain). Two different coupling agents, namely (3-aminopropyl)trimethoxysilane (APTMS) and (3-glycidoxypropyl)trimethoxysilane (GLYMO) were used to improve fibre-matrix interactions. Both coupling agents were supplied by Sigma Aldrich Spain (Madrid, Spain) and were selected because of both amine and glycidyl functionalities can readily react with the epoxy resin and hardener during the crosslinking process. On the other hand, the hydrolyzed alkoxy groups can react with hydroxyl groups in cellulose fibre thus leading to a coupling phenomenon between the fibre and the epoxy matrix. Scheme 1 shows the chemical structure of both silanes. As binder, a partially biobased epoxy resin was supplied by Resineco under the tradename Greenpoxy 55 with its corresponding hardener GP 505. The recommended resin:hardener was 100:40 (by weight) or 2:1 (by volume). This partially biobased epoxy has a renewable content of at least 55 wt % (55 wt % of carbon comes from plant, vegetables and/or oils) and possesses a density of 1.15 g cm^−3^, it is not soluble in water and its viscosity is 2000 cps at 25 °C.

### 2.2. Surface Treatments on Posidonia oceanica

PO fibres with a length in the 2–8 mm range were washed in distilled water with the main aim of removing residual sand, soil, etc. This operation was repeated until the water was clean enough. Then, the fibres were dried at 60 °C for 24 h in an air circulating oven Carbolite model PN (Hope Valley, UK). Some of these fibres did not receive any other treatment and were used as control. Some other dried fibres were subjected to an alkali treatment in an alkali water bath (5% NaOH) for 24 h with constant stirring. After this, the fibres were removed from the alkali bath, drained, washed with distilled water and subsequently dried at 60 °C for 24 h. An additional silanization treatment was carried out on alkali-treated PO fibres. Alkali treated fibres were placed in a water:acetone bath (50:50 *v*/*v*) containing 1% by weight of the corresponding silane and subjected to magnetic stirring for 2 h. After this treatment time, the silanized fibres were removed from the bath, drained and dried at room temperature for 48 h. 

### 2.3. Manufacturing of Fibreboards

Four different fibreboards were manufactured by hot-press moulding with untreated PO fibres (untreated), alkali treated fibres (NaOH) and alkali-treated fibres + silanization (labelled as NaOH + APTMS and NaOH + GLYMO). All composites contained a constant binder amount of 30 wt %. Initially, the fibres and the binder (resin + hardener) were weighed and mechanically mixed to homogenization. Due to the low density and high porosity of the PO fibres, all the resin was absorbed by the fibres during the mixing. Then, the mixtures were dropped into an aluminium mould with a cavity of 5 × 7 cm^2^ and placed in a hot plate press from DUPRA S.L. (Castalla, Spain). Then a pressure of 22–23 MPa was applied for 20 min to ensure full curing at 85 °C. Then, the temperature was switched off and a holding pressure of 22–23 MPa was maintained for an additional hour. After this, the moulded fibreboards with an average thickness of 4–5 mm were obtained and cut for sample characterization.

### 2.4. Mechanical Characterization

Flexural properties of the manufactured fibreboards were obtained in a universal test machine ELIB 30 from S.A.E. Ibertest (Madrid, Spain). Samples with an average size of 15 × 70 × 4.5 mm^3^, were subjected to a three-point bending flexural test as indicated by ISO 178 with a crosshead speed of 2 mm·min^−1^. At least five different samples were tested and the average values of the flexural strength (*σ_f_*) and modulus (*E_f_*) were calculated following Equations (1) and (2) respectively:

(1) $$\sigma_{f}=\frac{3FL}{2bh^{2}}$$

where *F* stands for the maximum flexural force in N, *L* is the distance between supports in mm, *b* is the width of the sample in mm and *h* is the height expressed in mm.

(2) $$E_{f}=\frac{\sigma_{f2}-\sigma_{f1}}{\varepsilon_{f2}-\varepsilon_{f1}}$$

where *σ_fi_* and *ε_fi_* stand for the flexural stress and elongation at two different points in the linear region. The flexural stresses are calculated by following Equation (1) and the corresponding elongations are calculated with Equation (3).

(3) $$s_{i}=\frac{\varepsilon_{fi}L^{2}}{6h}$$

where *s_i_* stands for the deflection and *ε_fi_* is the corresponding elongation.

The impact-absorbed energy of the fibreboards was obtained by using a 1 J Charpy’s pendulum from Metrotec following the guidelines of ISO 179:1993. Five different samples were tested in impact conditions and the average impact resistance was calculated from the absorbed-energy and the corresponding cross-section areas. In addition, the Shore D hardness of the manufactured fibreboards was obtained in a Shore D durometer from J. Bot Instruments (Barcelona, Spain). Each Shore D value corresponds to the average of, at least, five different measurements in different representative areas.

Dynamic-mechanical thermal characterization (DMTA) was carried out in an oscillatory rheometer model AR-G2 from TA Instruments (New Castle, DE, USA). This rheometer is equipped with a special clamp system for solid samples and it is possible to carry out dynamic tests in shear-torsion mode. The samples, with a size of 10 × 40 × 4 mm^3^ were subjected to a temperature ramp from 35 °C up to 120 °C at 2 °C·min^−1^, under a maximum deformation (%γ) of 0.1%. The frequency was set to 1 Hz. The most relevant dynamic properties, i.e., storage modulus (G′) and dynamic damping factor (tan δ) were collected.

### 2.5. Morphology of Composite Fibreboards

The fibreboards were morphologically characterized at two levels. Optical images of both the surface/appearance and fractures (from impact tests) were collected in a stereoscopic microscope Olympus model SZX7 (Barcelona, Spain) with a maximum magnification of 56×. Detailed images of fractured samples from impact tests were observed by scanning electron microscopy (SEM) in a Phenom microscope from FEI Company (Eindhoven, The Netherlands) at an acceleration voltage of 5 kV. Samples were subjected to a metallization process with gold in a sputter-coater Emitech SC7620 from Quorum Technologies (East Sussex, UK).

## 3. Results and Discussion

**Figure 2:**
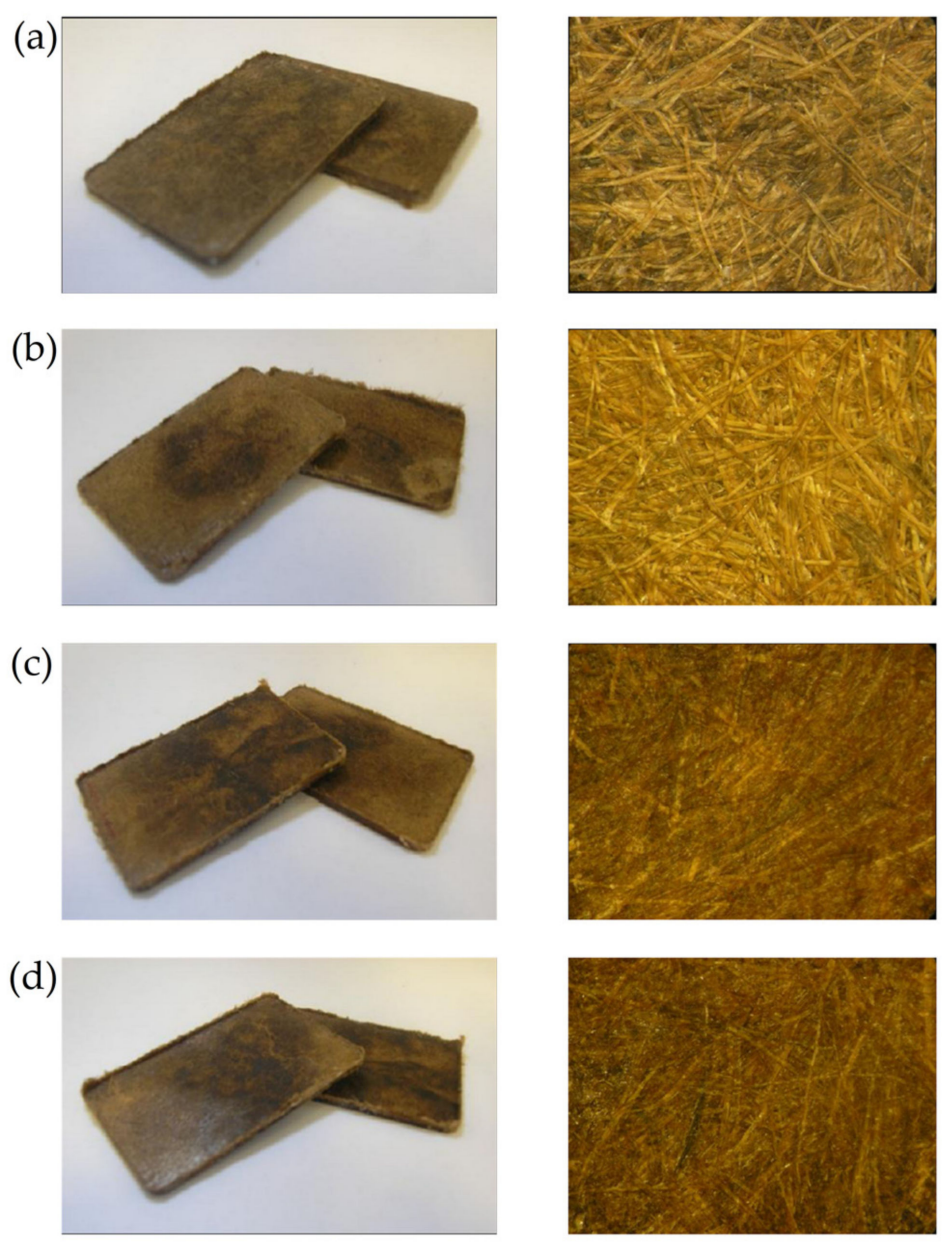
Optical images of the fibreboards (**left**) and the surface appearance (**right**) with PO fibres subjected to different chemical treatments, (**a**) untreated; (**b**) NaOH; (**c**) NaOH + APTMS and (**d**) NaOH + GLYMO.

All composite boards show a wood-like finish appearance as can be seen in Figure 2. This appearance is interesting as these composite fibreboards could find final applications in the building industry without any other surface finishing. As can be observed in Figure 2, there is not a preferential orientation of the fibres. PO fibres are randomly dispersed in the biobased epoxy matrix, leading to quasi-isotropic behaviour.

**Figure 3:**
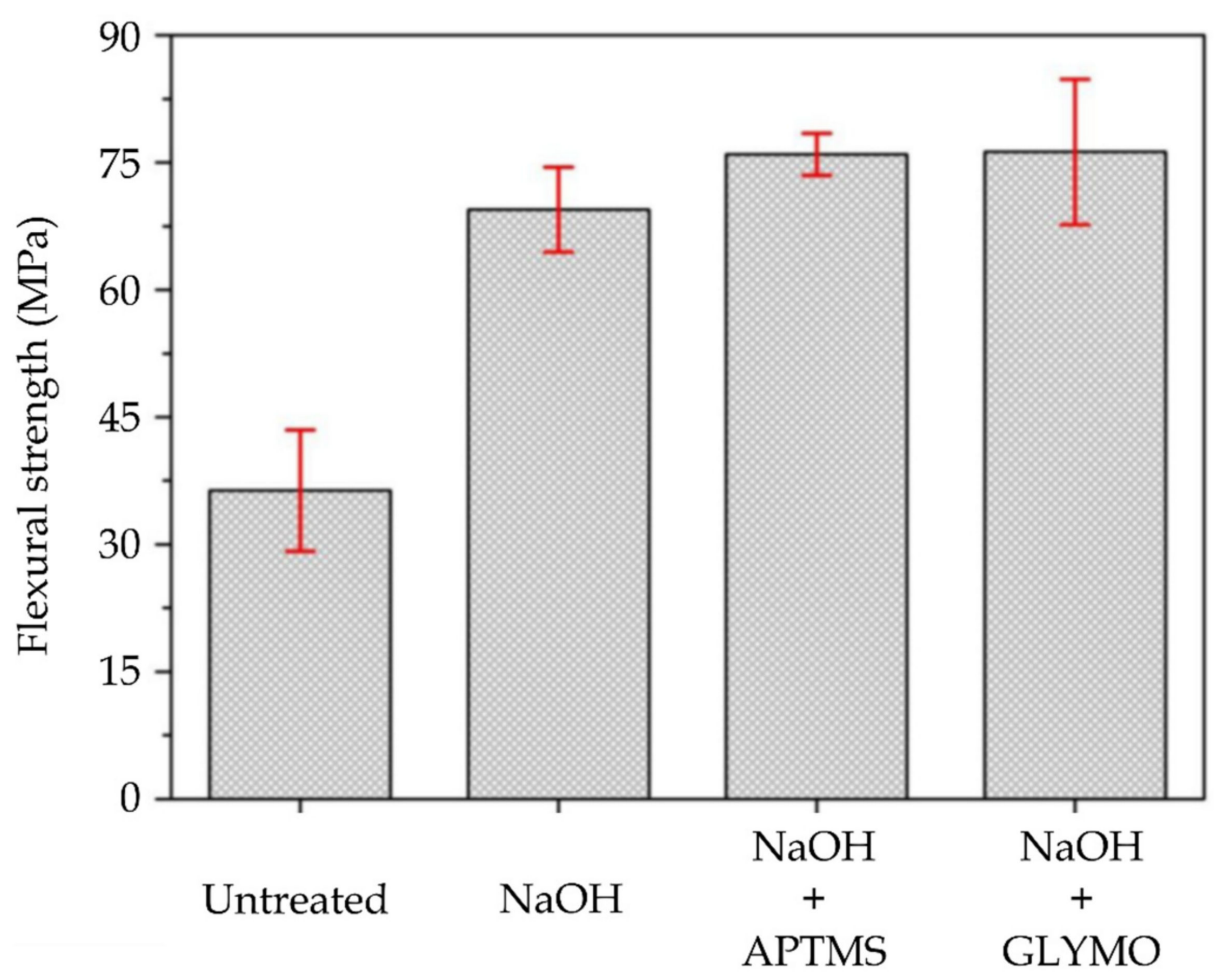
Bar plot of the flexural strength of PO fibreboards with different surface treatments.

Regarding mechanical performance, Figure 3 shows a plot representation of the flexural strength of the PO fibreboards with different surface treatments. Due to the non-preferential fibre orientation, these fibreboards show quasi-isotropic behaviour, since the fibres are randomly oriented. The flexural strength of the neat resin is 73 MPa. As can be seen, the flexural strength of fibreboards with untreated PO fibres is close to 36.4 MPa, which is remarkably lower than the neat resin. This is due to the lack of (or very poor) interaction between the fibre and the epoxy resin. It is worth noting the remarkable increase in flexural strength that a simple alkali treatment can provide. In fact, the flexural strength improves up to values around 70 MPa. The alkali treatment promotes a change in the morphology of PO fibres which leads to an increase in the surface roughness due to the removal of some fibre components such as lignin, hemicelluloses, impurities and other extractives [41]. With this, the α-cellulose content increases, as indicated by Jayaramudu et al. [42]. This has a positive effect on fibre/matrix interactions that allows better load transfer from the matrix to the fibre. G. Goud et al. reported a remarkable increase in flexural and tensile strength on epoxy composites with royal palm fibre subjected to alkali treatment [43]. After the alkali treatment, they reported an increase in the α-cellulose content from 58% to 65% with the subsequent increase in mechanical performance. In addition, the alkali treatment also promotes the breakage of the fibre bundles into smaller fibres, all this having a positive effect on fibre-matrix interactions due to increased wetting surface [44,45]. As can be seen in Figure 3, a silanization treatment after a previous alkali treatment also enhances the mechanical performance of PO fibreboards. Specifically, the flexural strength increases up to values of 76 MPa, which represents a percentage increase of almost 101% with regard to fibreboards with untreated PO fibres, thus showing the efficiency of the combined alkali + silanization surface treatment. These results are in total agreement with those reported by Zhu et al. with flax-fibre-based composites, a different polymer matrix and different surface treatments, i.e., mercerization, silanization, acylation and peroxide treatments to increase particle-polymer interaction [46]. Nishitani et al. also reported the positive effect of silane coupling agents in polyamide 1010/hemp fibre composites showing an improved tribological response due to increased polymer-reinforcement interaction [47].

**Figure 4:**
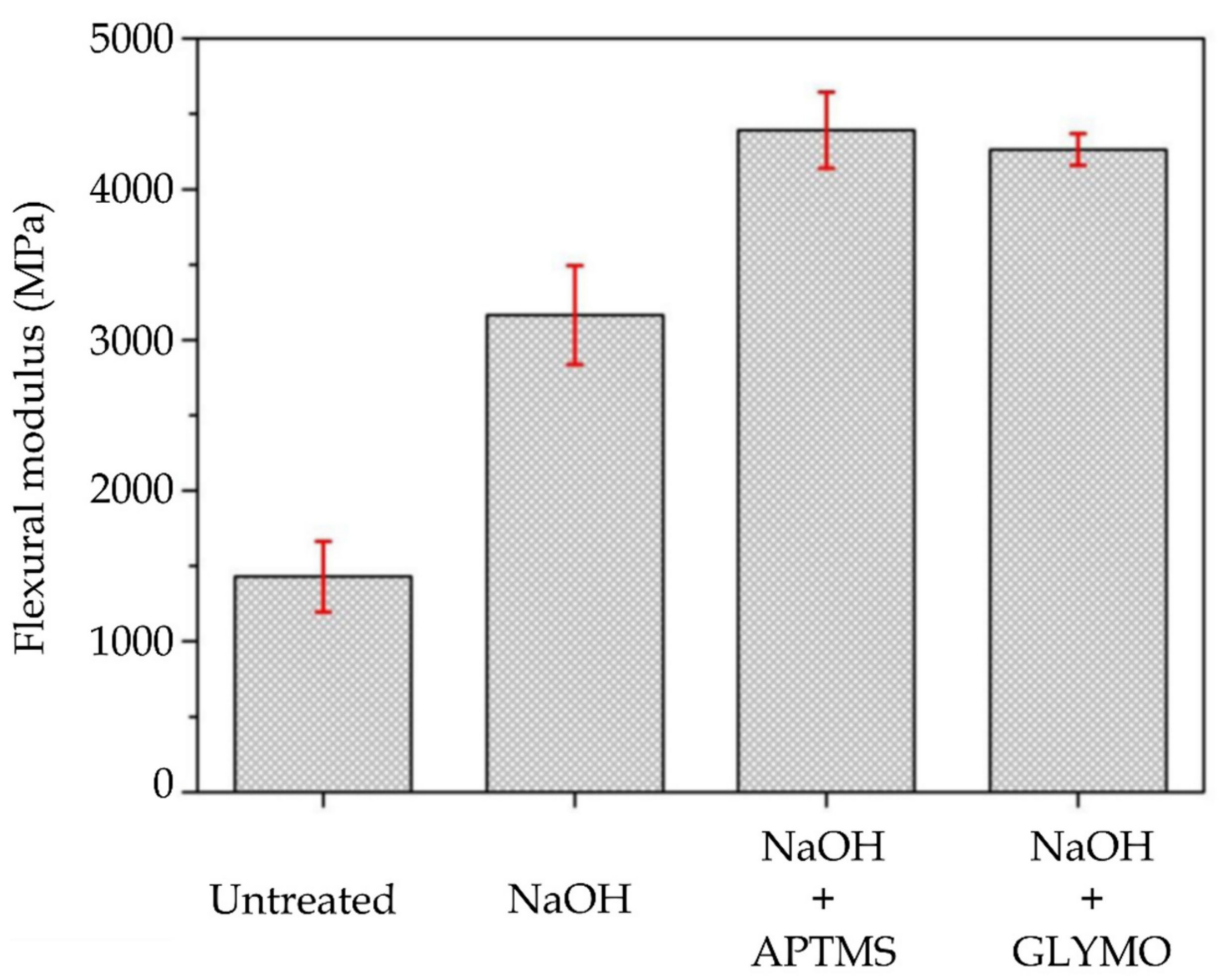
Bar plot of the flexural modulus of PO fibreboards with different surface treatments.

Similar effects can be observed for the flexural modulus (Figure 4). If we take into account the flexural modulus of the neat epoxy resin, which is around 2.0 GPa, fibreboards with untreated PO fibres offer a lower flexural modulus of 1.4 GPa that can be ascribed to the above-mentioned poor fibre-matrix interaction. As it can be observed, the flexural modulus changes from 1.4 GPa (fibreboards with untreated PO fibres) up to 4.2–4.3 GPa for both silane-treated PO fibreboards. With regard to the alkali treatment, all the above-mentioned effects lead to a flexural modulus of about 3.2 GPa, thus showing the efficiency of the alkali treatment itself without additional treatments. Nevertheless, the obtained results suggest a synergistic effect with the additional silanization treatment, which leads to almost three times higher flexural modulus regarding the fibreboards with untreated PO fibres. In general, the flexural properties of the neat resin are improved, since the flexural strength remains at similar values of those of the neat resin, and the flexural modulus increases in a remarkable way.

**Figure 5:**
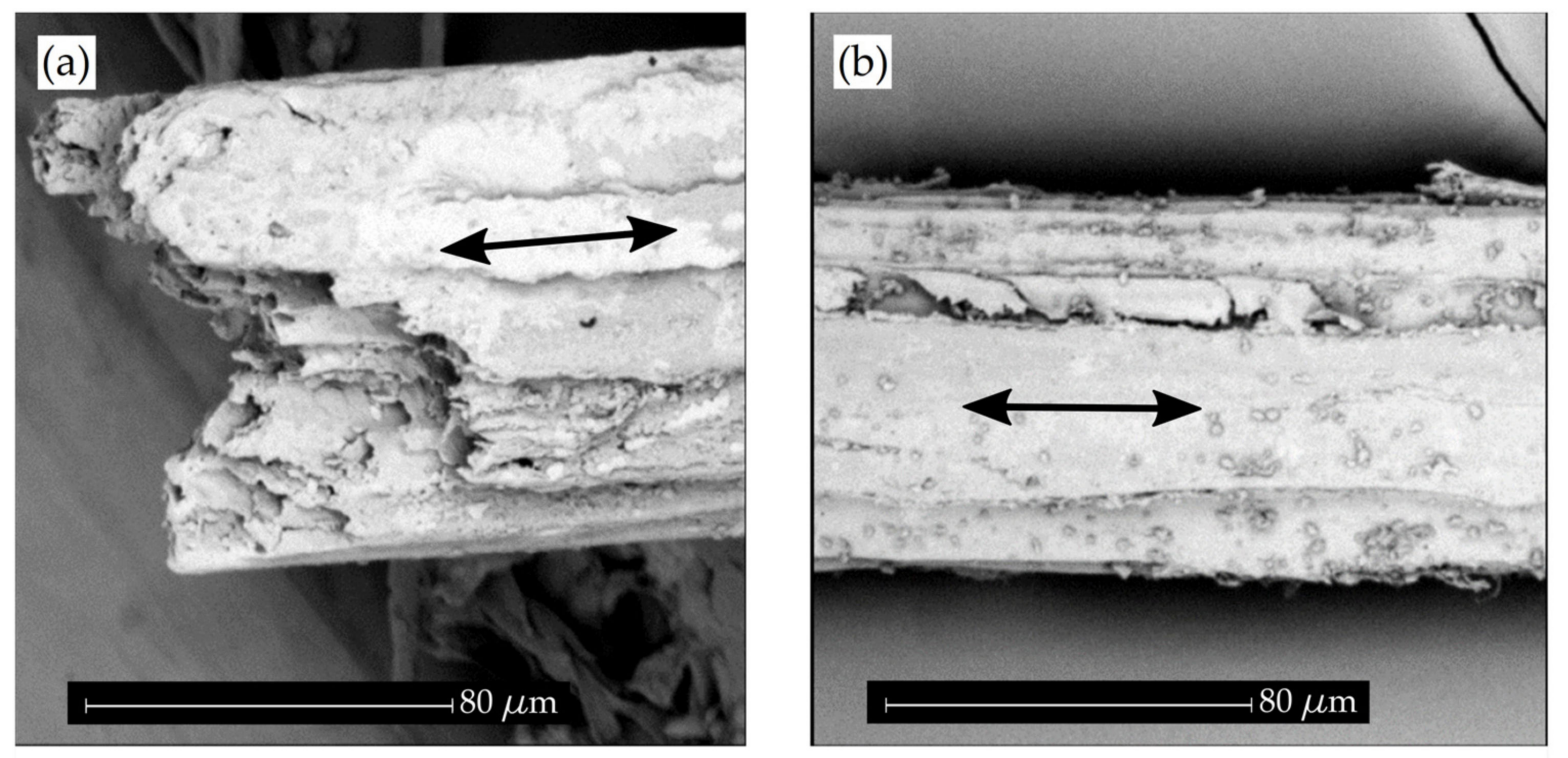

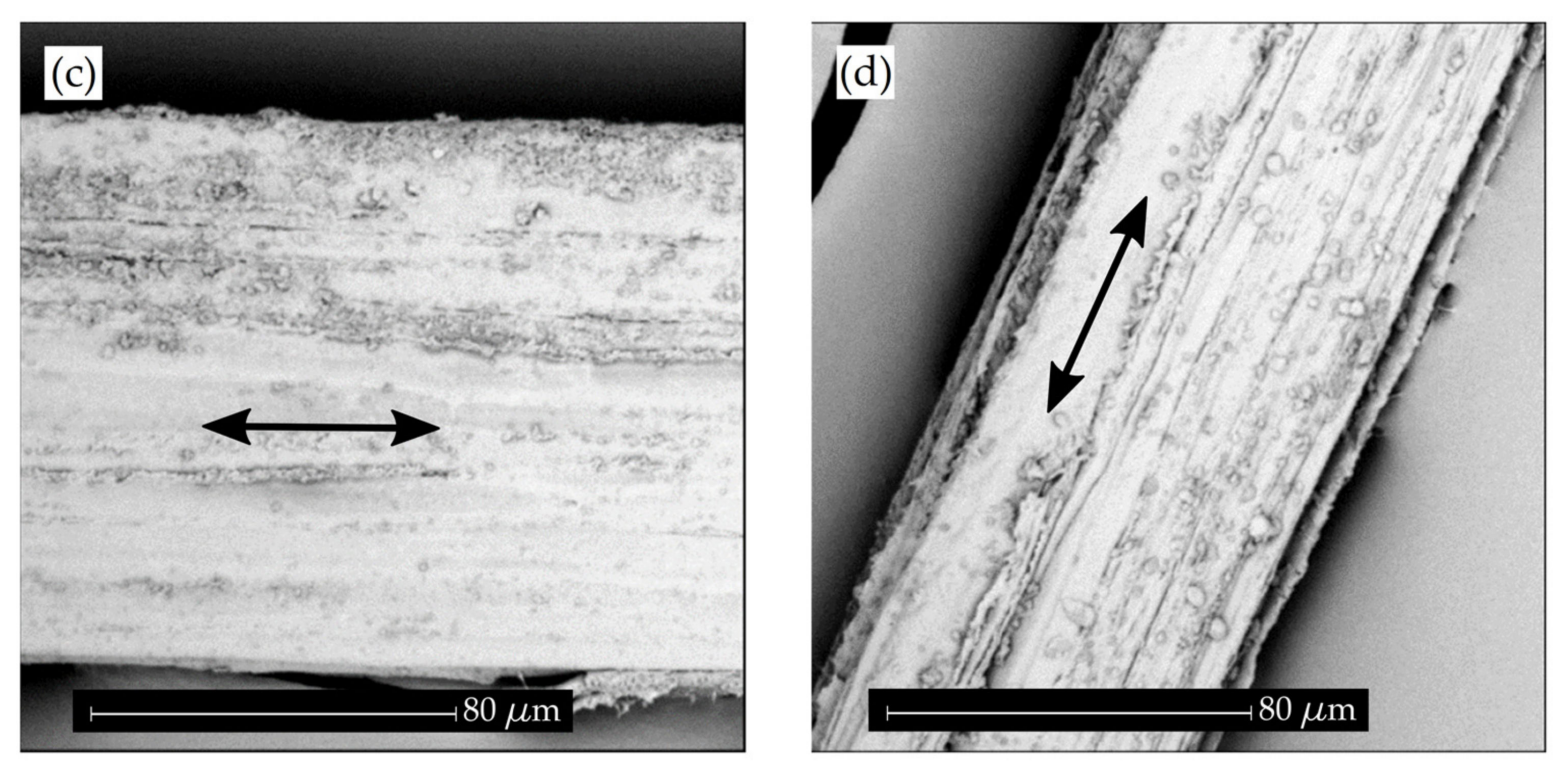
SEM images at 1500× longitudinal PO fibres subjected to different chemical treatments, (**a**) untreated; (**b**) NaOH; (**c**) NaOH + APTMS and (**d**) NaOH + GLYMO (black arrows indicate the fibre axis).

**Scheme 2:**
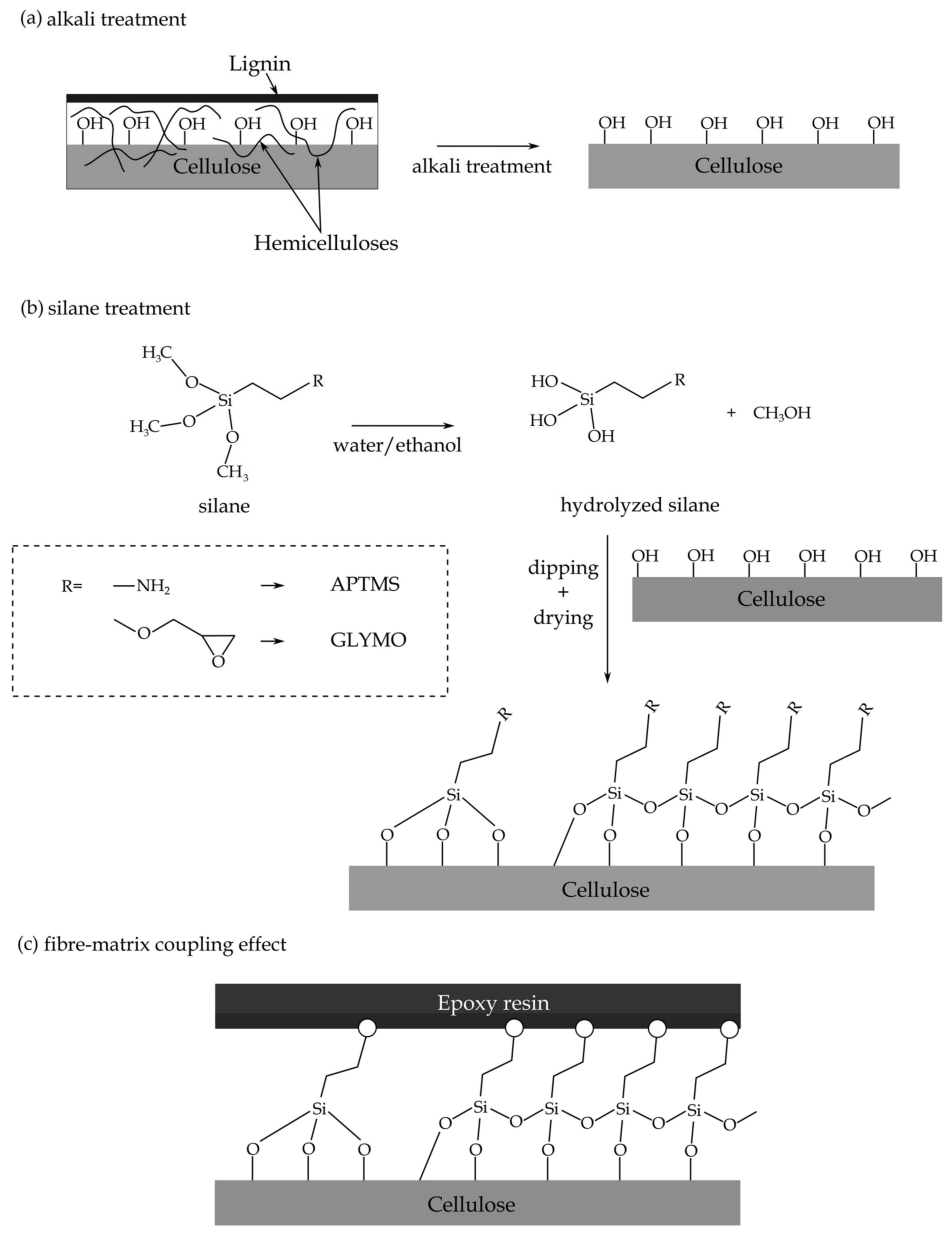
Schematic representation of the effect of alkali and silane treatment on a natural fibre surface and a possible coupling mechanism between PO-treated fibres and epoxy resin.

These results are directly related to the surface treatment. Figure 5 shows the morphology of PO fibres with different surface treatments. The alkali treatment promotes the removal of lignin, hemicelluloses and other compounds which leads to a slight change in roughness (Figure 5b). With regard to the silanized PO fibres, the previous alkali treatment allows better interactions between the hydroxyl groups in cellulose and the hydrolyzed silane structure that could lead to a chemical bonding with the fibre surface as shown in Scheme 2. This bonding plays a key role in acting as a bridge between the PO fibres and the epoxy binder, with the subsequent improvement of mechanical performance. Figure 5c,d show SEM images of silanized PO fibres after the above-mentioned alkali treatment. The most visible feature that silanes provide to the treated fibres is surface homogeneity, since an ultrathin homogeneous layer is chemically linked to the readily available hydroxyl groups in cellulose after the extraction of lignin and hemicellulose by the alkali treatment. Nevertheless, the changes in surface roughness are not remarkable, but silanized fibres allow better interactions with the partially biobased epoxy resin as mechanical properties suggest. 

As per the results, both the amino- and the glycidyl-silane seem to give a similar mechanical performance based on flexural behaviour. This can be explained by assuming that both functional groups can react with the epoxy resin, thus leading to an additional coupling effect between PO fibres and the epoxy binder, as can be observed in Scheme 2. Although the alkali treatment is enough to give improved mechanical performance (mainly to increase roughness derived from lignin and hemicellulose extraction), hydrophilicity is high since after this treatment, the hydroxyl groups of cellulose are more readily available [48]. Silanes are characterized by dual functionality. On the one hand, silanes have one or several alkoxi groups and, in general, an additional organic functionality such as epoxy, amine, mercaptane, acrylic acid, vinyl, etc. When silanes are hydrolyzed, the alkoxy groups are converted into hydroxyl groups which can readily react with the hydroxyl groups in cellulose to for an ultrathin silane layer chemically bonded to the cellulose component. The other functional group, i.e., amine or glycidyl in this work, does not take part in this reaction with cellulose and remains free to react with the thermosetting resin (both the epoxy resin and the hardener). This allows a chemical link between the silane and the epoxy resin. The overall effect of the silane is a chemical attachment to both the cellulose fibre and the epoxy resin, thus acting as a bridge between them and, subsequently, improving fibre-matrix interactions.

**Figure 6:**
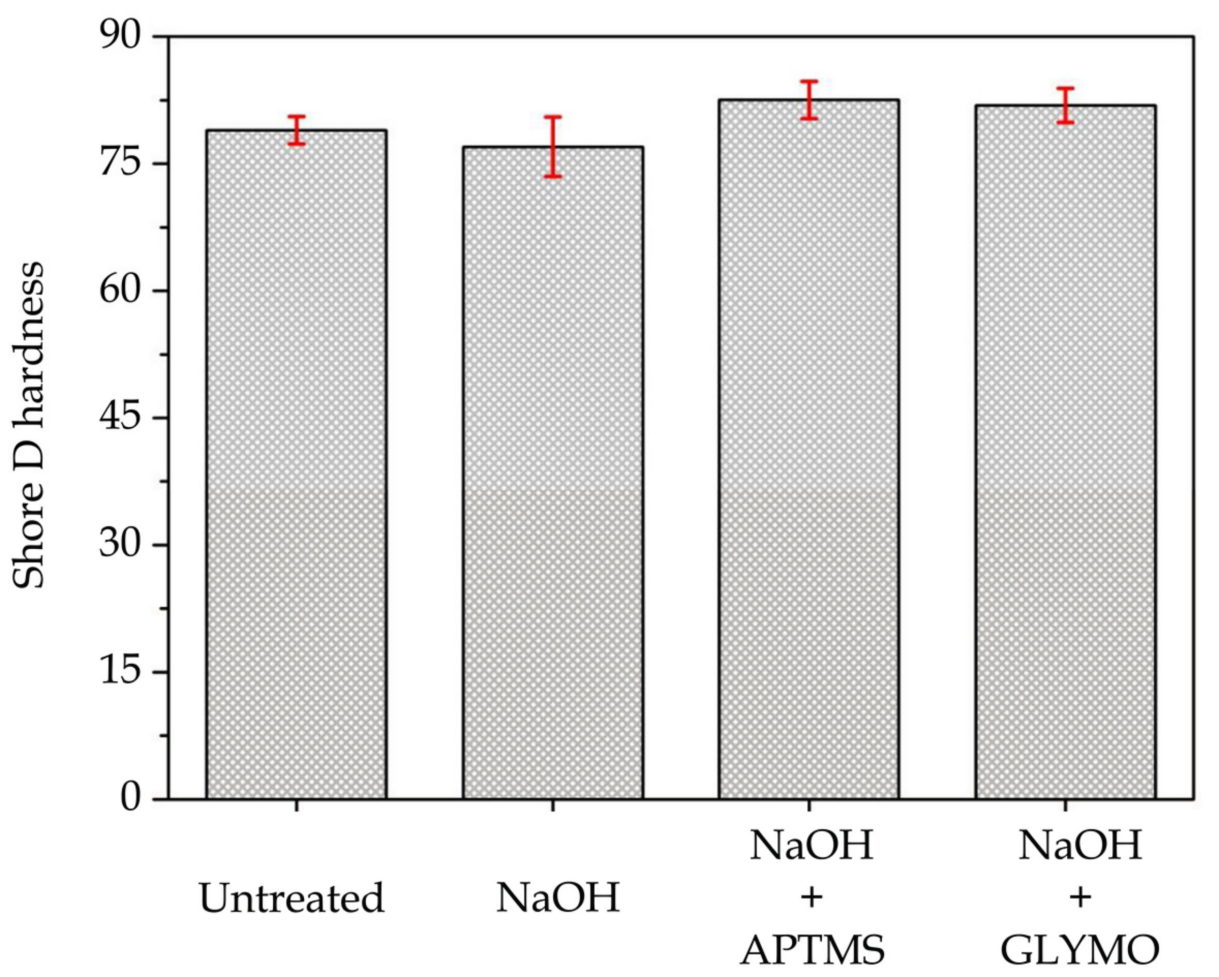
Bar plot of the hardness (Shore D) of PO fibreboards with different surface treatments.

**Figure 7:**
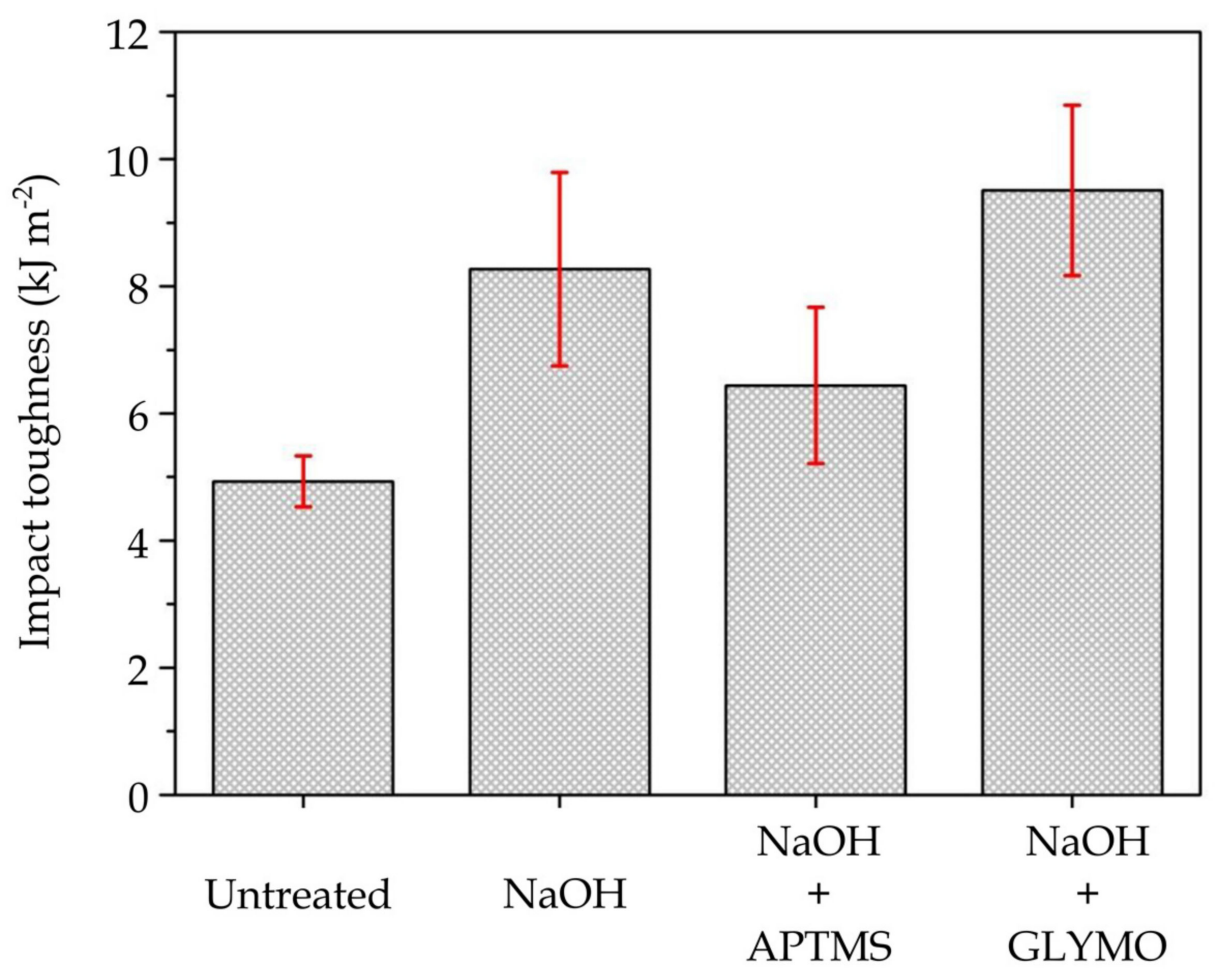
Bar plot of the impact toughness (Charpy test) of PO fibreboards with different surface treatments.

With regard to the hardness, the Shore D values do not show significant changes, as can be seen in Figure 6, despite the fact that slight variations can be detected. In fact, slightly lower values are obtained for the alkali-treated fibreboard and slightly higher values are observed for the silanized fibreboards. It is in the impact-absorbed energy that important differences can be detected, as observed in Figure 7. The fibreboards with untreated PO fibres are characterized by a relatively low impact resistance due to low fibre/matrix interactions. This leads to an impact resistance of less than 5 kJ·m^−2^. The only alkali treatment leads to improved impact properties with values of about 8.3 kJ·m^−2^, but it is the glycidyl silane treatment which gives the best impact properties, with values of more than 9 kJ·m^−2^, thus showing its higher efficiency compared to the alkali and the alkali + amino silane treatment. The impact-absorbed energy is directly related to the silane chemical structure and its ability to react with different components. The amino silane offers a shorter carbon chain than the glycidyl silane. For this reason, the longer glycidyl silane chain can be stretched to a greater extent than the shorter amino silane chain, so that the glycidyl silane leads to increased toughness. On the other hand, the amine group in APTMS can directly react with epoxy groups in the resin with a similar effect to a typical amine hardener, and this leads to a short chain bridge between the cellulose and the epoxy. On the other hand, the glycidyl group in GLYMO can react with the hardener (an amine-type) which, in turn, can react with epoxy groups in the epoxy resin, thus leading to a longer bridging chain which contributes to improved toughness.

**Figure 8:**
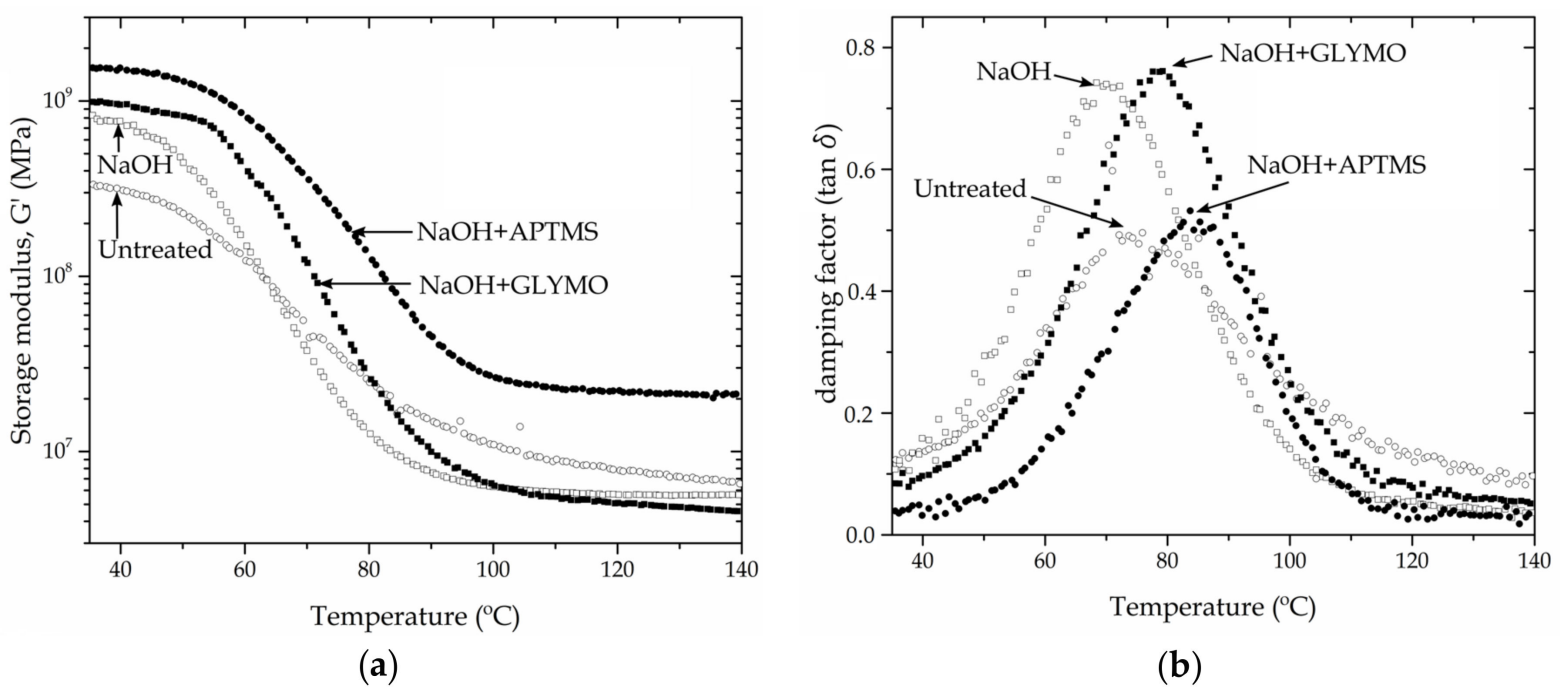
Plot evolution of the (**a**) storage modulus (G′) and (**b**) damping factor (tan δ) in function of temperature for fibreboards with PO subjected to different surface treatments.

Regarding the dynamic mechanical behaviour of PO fibreboards, Figure 8 gathers the evolution of the storage modulus (G′) and the damping factor (tan δ) in terms of increasing temperature for the different treatments. The composite with untreated PO fibres shows the lowest G′ value (344.7 MPa at 35 °C) (Figure 8a). All three surface treatments promote an increase in the storage modulus in a similar way as previously described for the flexural modulus. It is worth noting that the maximum G′ value was achieved for PO fibreboards with NaOH + APTMS silane treatment with values of about 1 GPa at 35 °C. These results are in total agreement with the above-mentioned results for flexural properties. By considering the glass transition temperature (T_g_) at the peak value of the damping factor, it is possible to conclude that silanes exert a direct effect on T_g_, as can be seen in Figure 8b. The fibreboard with untreated PO fibres shows a T_g_ located around 74 °C. The alkali treatment with NaOH does not lead to a remarkable change in T_g_. Nevertheless, both silanes (amino and glycidyl) produce a noticeable increase in the T_g_, reaching values of 83.7 °C and 79.2 °C for the amino- and glycidyl-silane respectively. This indicates good coupling effects between the PO fibres and the binder epoxy resin. The glass transition temperature is increased, as can be seen in Table 1. A direct relationship has been reported between the toughness and the damping factor. As the toughness increases the damping peak (and more specifically, the loss area) increases due to energy absorption. This situation can be seen in Figure 8 as the loss area is higher for all treated fibreboards, thus indicating that the effect on toughness is positive [49].

**Figure 9:**
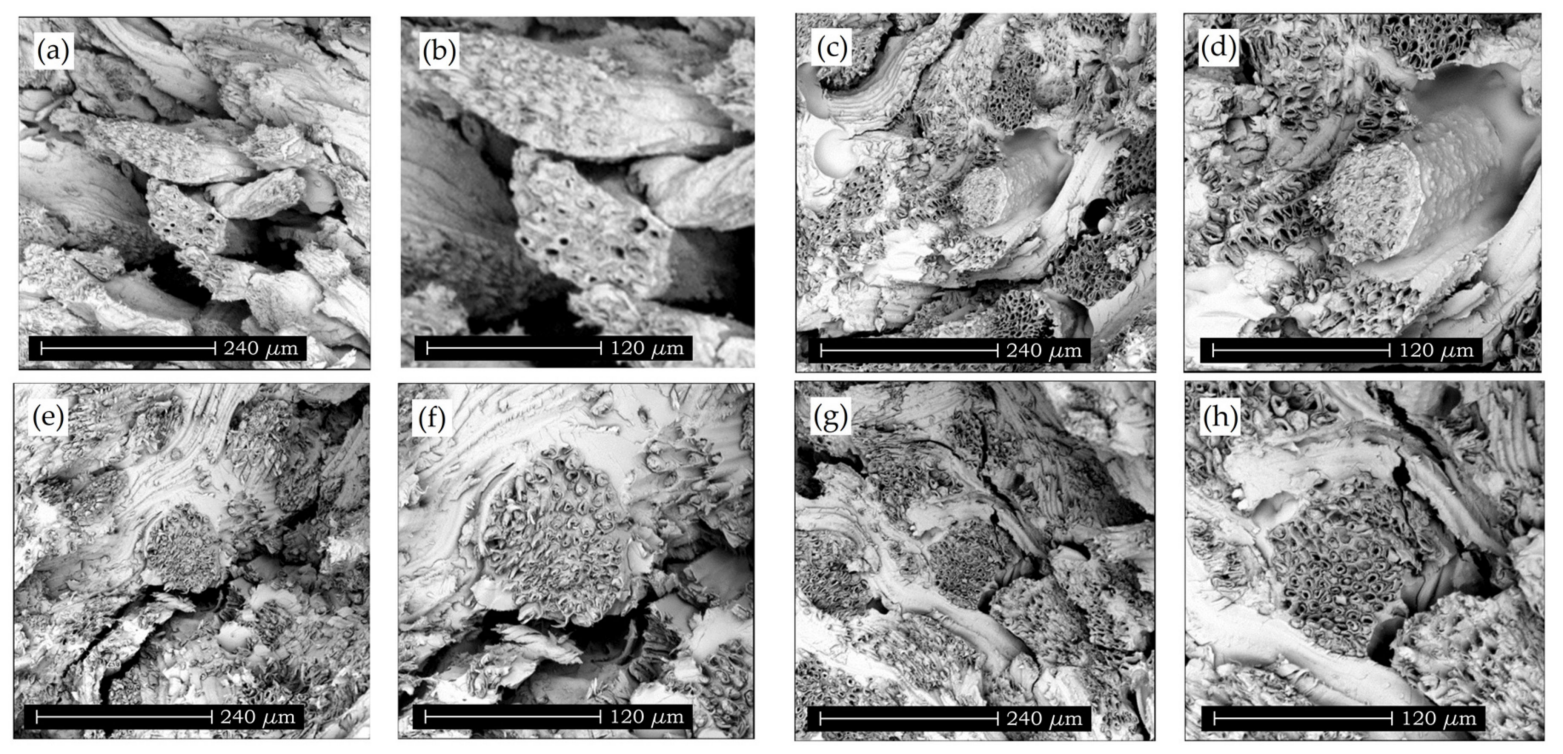
SEM images at different magnifications (a, c, e, g taken at 500×; b, d, f, h taken at 1000×) of the fractured surface of fibreboards with PO fibres subjected to different chemical treatments, (**a**,**b**) untreated; (**c**,**d**) NaOH; (**e**,**f**) NaOH + APTMS and (**g**,**h**) NaOH + GLYMO. Black arrows show the improved wetting behaviour between the PO fibres and the surrounding epoxy resin.

The morphology of PO fibreboards has been analysed by SEM to elucidate the potential fibre-matrix interactions. This partially biobased epoxy offers better wetting properties towards natural fibres than petroleum-derived epoxies, even without any surface treatment, as reported by Bertomeu et al. [50]. Figure 9 gathers SEM images at different magnifications corresponding to fractured samples of PO fibreboards from impact tests. Figure 9a,b correspond to composite fibreboards with untreated PO fibres. The lack of interaction between the PO fibres and the surrounding epoxy matrix is clearly observable. PO fibres do not interact with the epoxy resin and this leads to poor mechanical performance, as the loads cannot be transferred in an appropriate way. In addition, the SEM images suggest that the PO fibres are not wet by the epoxy resin. This situation is quite different in fibreboards with only an alkali treatment (Figure 9c,d). As can be seen, fibreboards with alkali-treated PO fibres somewhat show fibre-matrix interactions that are responsible for the increase in mechanical performance, as has been previously described. The black arrows in Figure 9d are representative of areas with increased wettability, as it seems that the PO fibre is covered by an epoxy layer. The superior effect of the silanized PO fibres can be observed in Figure 9e,f (NaOH + APTMS) and Figure 9g,h (NaHO + GLYMO). Both silanes produce similar morphologies, characterized by fully-embedded PO fibres in the epoxy matrix. Although some gaps can be detected, the overall effect of both silanes is a clear reduction of the gap between the PO fibres and the surrounding epoxy matrix, as emphasized by the black arrows in Figure 9f,h, corresponding to fibreboards with PO fibres treated with NaOH + APTMS and NaOH + GLYMO, respectively.

## 4. Conclusions

This work reports the development of highly environmentally-friendly composite fibreboards with *Posidonia oceanica* (PO) waste seagrass as the lignocellulosic component. Fibreboards with a constant fibre content of 70 wt % PO were manufactured by hot-press moulding using a partially biobased epoxy binder resin, leading to fibreboards with more than 85 wt % biobased content. The obtained materials showed a wood-like appearance. The mechanical properties of untreated fibreboards were relatively poor due to the lack of interaction between the lignocellulosic component and the epoxy matrix. For this reason, several treatments were proposed, i.e., alkali treatment with NaOH and alkali treatment followed by silanization with different silanes (amine silane, APTMS, and glycidyl silane, GLYMO). Although a simple alkali treatment improved the mechanical performance, the use of an alkali treatment followed by silanization led to a remarkable increase in the mechanical behaviour of PO fibreboards. In particular, the flexural strength and modulus were remarkably improved and, interestingly, the impact toughness was noticeably increased due to the coupling effect that silanes can provide. The SEM study revealed increased fibre-matrix interaction, as the gap between them was remarkably reduced or even eliminated. In general, these fibreboards represent an interesting alternative to the conventional natural fibre particle of fibreboards, as the used binder also offers high environmental efficiency.

## Figures and Tables

**Table 1:** Storage modulus (G′) values at different temperatures and glass transition temperatures (T_g_) for fibreboards with PO subjected to different surface treatments.

Surface Treatment	G′ (MPa) at 35 °C	G′ (MPa) at 100 °C	T_g_ (°C)
Untreated	344.7	11.0	73.7
NaOH	808.6	6.6	70.0
NaOH + APTMS	1551.0	26.4	83.7
NaOH + GLYMO	1014.0	6.3	79.2
